# Construction of a predictive model of 2–3 cm ground-glass nodules developing into invasive lung adenocarcinoma using high-resolution CT

**DOI:** 10.3389/fmed.2024.1403020

**Published:** 2024-06-21

**Authors:** Yifan Zhang, Lin Qu, Haihua Zhang, Ying Wang, Guizhou Gao, Xiaodong Wang, Tao Zhang

**Affiliations:** ^1^Department of Thoracic Surgery, Tangdu Hospital, Air Force Medical University, Xi’an, China; ^2^Department of Respiratory Medicine, Tangdu Hospital, Air Force Medical University, Xi’an, China

**Keywords:** GGN, HRCT, risk factors, nomogram, lung cancer

## Abstract

**Background:**

The purpose of this study was to analyze the imaging risk factors for the development of 2–3 cm ground-glass nodules (GGN) for invasive lung adenocarcinoma and to establish a nomogram prediction model to provide a reference for the pathological prediction of 2–3 cm GGN and the selection of surgical procedures.

**Methods:**

We reviewed the demographic, imaging, and pathological information of 596 adult patients who underwent 2–3 cm GGN resection, between 2018 and 2022, in the Department of Thoracic Surgery, Second Affiliated Hospital of the Air Force Medical University. Based on single factor analysis, the regression method was used to analyze multiple factors, and a nomogram prediction model for 2–3 cm GGN was established.

**Results:**

(1) The risk factors for the development of 2–3 cm GGN during the invasion stage of the lung adenocarcinoma were pleural depression sign (OR = 1.687, 95%CI: 1.010–2.820), vacuole (OR = 2.334, 95%CI: 1.222–4.460), burr sign (OR = 2.617, 95%CI: 1.008–6.795), lobulated sign (OR = 3.006, 95%CI: 1.098–8.227), bronchial sign (OR = 3.134, 95%CI: 1.556–6.310), diameter of GGN (OR = 3.118, 95%CI: 1.151–8.445), and CTR (OR = 172.517, 95%CI: 48.023–619.745). (2) The 2–3 cm GGN risk prediction model was developed based on the risk factors with an AUC of 0.839; the calibration curve *Y* was close to the *X*-line, and the decision curve was drawn in the range of 0.0–1.0.

**Conclusion:**

We analyzed the risk factors for the development of 2–3  cm GGN during the invasion stage of the lung adenocarcinoma. The predictive model developed based on the above factors had some clinical significance.

## 1 Introduction

Studies have shown that lung cancer accounts for 2.2 million new cases and 1.79 million deaths annually and is the leading cause of cancer-related deaths worldwide (1, 2). Adenocarcinoma of the lung is the most common subtype of lung cancer. The early-stage adenocarcinoma of the lung is characterized by ground-glass-like, cloud-like, round, or irregular nodules, which are described as ground-glass nodules (GGN). According to statistics, approximately 9.1% of the population have GGN, and 4.95% of these nodules are diagnosed as malignant (3). Over the past decade, with the application of low-dose CT screening and high-resolution CT, an increasing number of early-stage lung adenocarcinoma cases with GGN were found, diagnosed, and treated surgically. The overall mortality rate of lung cancer has reduced by 26–61%, and this data suggest that early detection and early intervention are the most effective ways to improve lung cancer prognosis (4, 5).

**Figure 1:**
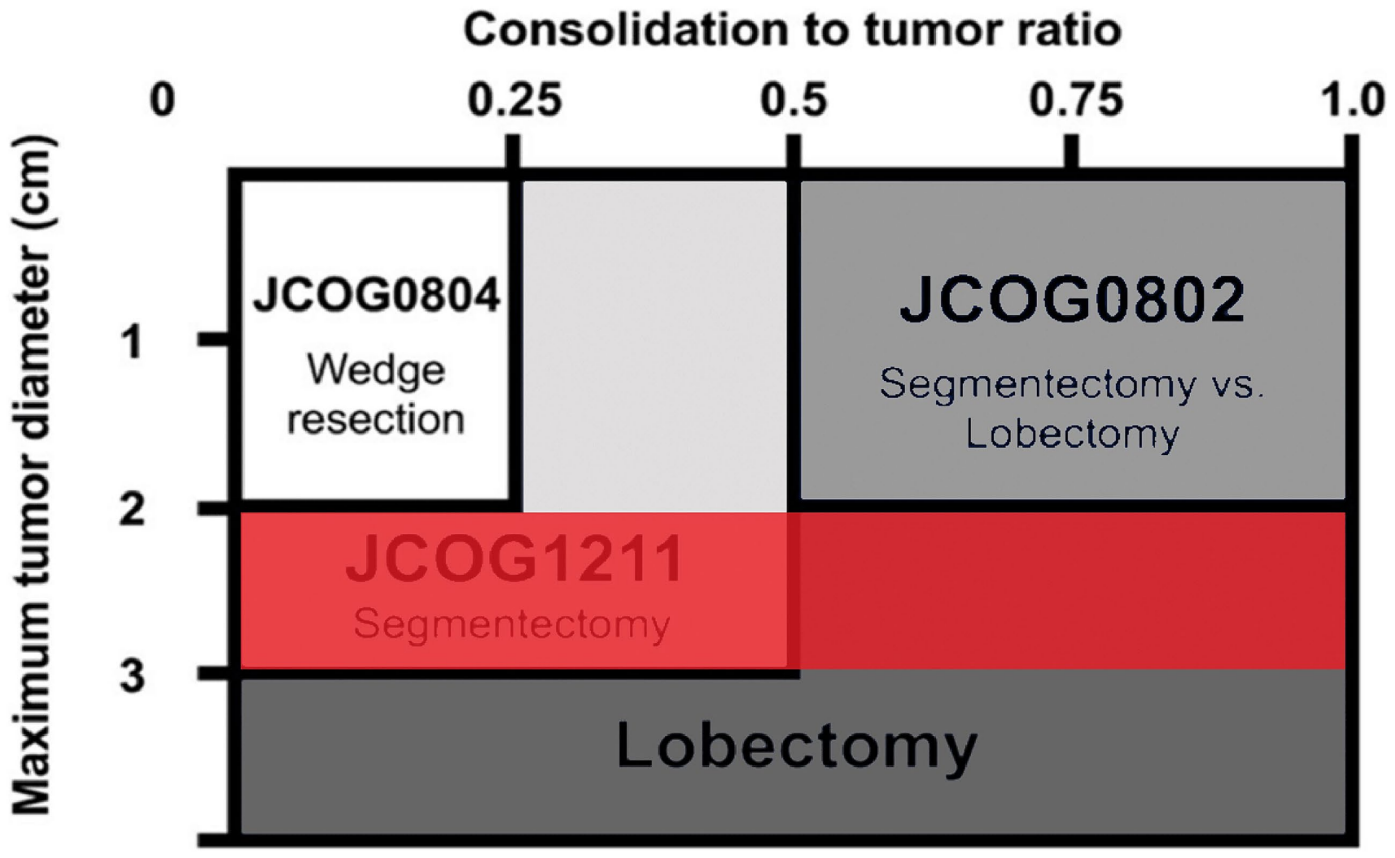
The three research schematic diagrams, 0804, 0802, 1211, and our research goals.

The development of lung adenocarcinoma undergoes four stages: Atypical Adenomatous Hyperplasia (AAH), Adenocarcinoma *in Situ* (AIS), microinvasive adenocarcinoma (MIA), and invasive adenocarcinoma (IAC) (6, 7). The GGN in different stages of development has different imaging characteristics, the corresponding intervention processing is also different. Several recent imaging studies utilizing a thin-slice CT have provided a good basis for classifying the different stages of development of malignant pulmonary nodules and address them accordingly (8–10). For example, three studies from the Japanese group of clinical oncology, 0802, 0804, and 1211 (Figure 1) (11–13), used the CTR value (GGN solid component diameter/maximum diameter) as the reference value. It is suggested that the standard lobectomy should be performed when the CTR value is >0.5, and sublobectomy should be performed when the CTR value is <0.5. It can be concluded that the imaging features of GGN in the invasive stage are of great significance in guiding the surgery. However, due to the large size of the nodules, 2–3 cm GGN showed more imaging features. The Japanese study, named 1211 used only CTR > 0.5 as the criterion for predicting the invasion stage. The imaging features of the vacuole sign, spiculation sign, lobulation sign, Vascular bundle sign, and the Bronchial sign, which had some defects, were neglected. Therefore, we intend to review the information, imaging features, and postoperative pathology of 2–3 cm GGN patients in our hospital from 2017 to 2022 and establish a predictive model, using multivariate analysis, for the development of 2–3 cm GGN infiltrative stage lung adenocarcinoma. This information will help guide the operation more effectively.

## 2 Materials and methods

### 2.1 Study design and participants

This retrospective study was performed at the Department of Thoracic Surgery, Second Affiliated Hospital of the Air Force Medical University. We enrolled 596 adults who underwent surgical resection of GGN during lung cancer surgery between January 2017 and December 2022.

#### 2.1.1 Inclusion criteria

Patients were 18–75 years of age, had a maximum tumor diameter of 2–3 cm, had no previous history of chemotherapy and/or radiotherapy for any malignant disease, had an expected forced expiratory volume of 800 mL in 1.0 s after surgery and partial pressure of oxygen (PaO_2_) over 65.

The intraoperative requirements were surgery within 28 days of the initial hospital stay, histologically proven non-small-cell lung carcinoma, absence of malignant pleural effusion, absence of pleural dissemination, absence of lymph node involvement, and the lesion could be removed surgically by lobectomy or segmental pneumonectomy with lymphadenectomy.

#### 2.1.2 Exclusion criteria

Patients with the presence of active bacterial or fungal infections; concurrent or metachronous (within the past 5 years) dual cancer; interstitial pneumonia, pulmonary fibrosis, or severe emphysema; psychosis; systemic steroid medications; poorly controlled diabetes; poorly controlled high blood pressure; or a second hospital stay with a history of serious heart disease.

The Ethics Committee of the Second Affiliated Hospital of the Air Force Medical University approved the study protocol. The study was conducted in accordance with the principles of the Declaration of Helsinki.

### 2.2 Data collection

General information, including sociodemographic status, family history, history of chronic respiratory disease, and smoking history (smoking years, daily smoking), was collected from enrolled patients.

HRCT: The primary HRCT report was written by Chen Zhuhong, the chief physician of the Imaging Department, and the HRCT was evaluated by Qin Xu, who is a deputy chief physician, and Cui Ming, who is a chief physician. The Associate Chief Physicians of Thoracic Surgery, Gao Guizhou, Xiaodong Wang, and Kühling cross-checked the reports and entered them into our data set. The imaging features included the pleural depression sign, vacuole sign, burr sign, lobulated sign, bronchial sign, diameter of GGN, and CTR.

### 2.3 Pathological diagnosis

The tumor tissue was delivered to the pathology department of our hospital for formaldehyde fixation within 2 h after resection. After 72 h, Hematoxylin and eosin (HE) staining and microscopic observation were performed to evaluate the pathological stages (AAH, AIS, MIA, and IAC) of the nodules. The pathological diagnosis was issued by the chief physician, Li Gong, and the deputy chief physician, Lan-lan Feng of the Department of Pathology.

### 2.4 Statistical analysis

Each factor was analyzed by univariate logistic analysis using SPSS 26.0 statistical software. A multivariate logistic analysis was performed using the back-stepping method to assess the risk factors for the development of invasive lung adenocarcinoma in 2–3 cm GGN and to calculate the odds ratios (OR). The model was adjusted for the pleural depression sign, vacuole sign, burr sign, lobulated sign, bronchial sign, diameter of GGN, and CTR. A *p*-value of <0.05 was considered statistically significant. An OR of >1.0 was considered to indicate a risk factor for the occurrence of 2–3 cm GGN infiltrative stage lung adenocarcinoma development, while an OR of <1.0 was considered to indicate a protective (preventive) factor against the occurrence of 2–3 cm GGN infiltrative stage lung adenocarcinoma development.

For the construction and validation of the nomogram, the subjects were randomly divided into a training set and a validation set at a ratio of 2:1. Following the multivariate analysis, factors with a two-sided *p*-value of <0.05 were selected to construct the nomograms. The predictive accuracy of the nomograms was estimated by the area under the ROC curve (AUC) of the receiver operating characteristic (ROC) curve in both the training and validation sets. The consistency between the actual outcomes and predicted probabilities was measured by the calibration curve. The clinical utility of the nomograms was determined by Decision curve analysis (DCA) and clinical impact curves for a sample size of 1,000.

## 3 Results

**Figure 2:**
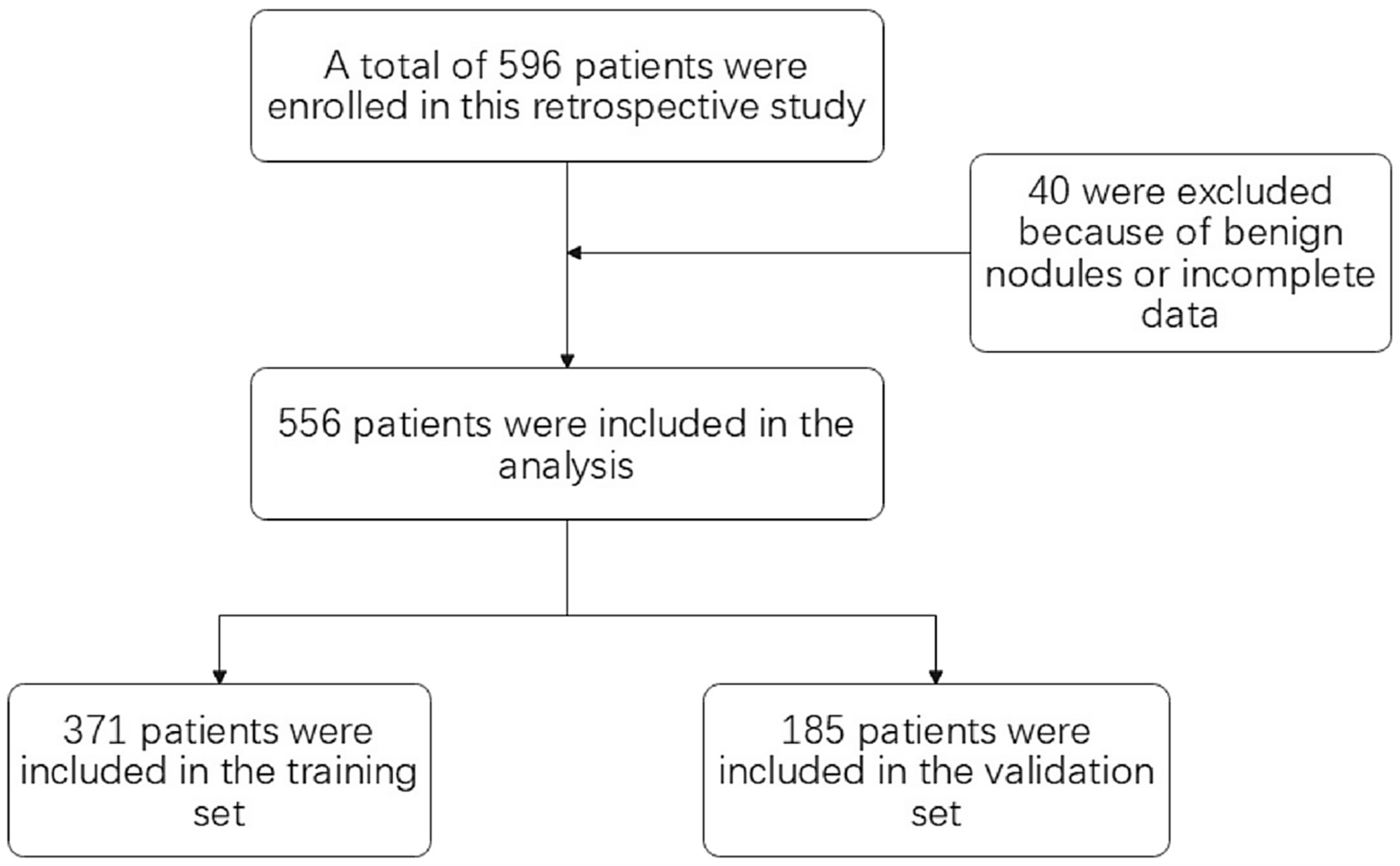
Flow of participants through the study.

Of the 596 adults enrolled, 40 adults were excluded because of benign nodules or incomplete data. A total of 556 adults were included in the study (371 in the training set and 185 in the validation set) (see Figure 2). The mean age of the participants was 61.51 years (SD of 26.51 years), and in our cohort, 77.89% (433 of 556 adults) of the study population had pathologically suggestive infiltrative periods. There were significant differences observed in the lobulated sign (*p*<0.001), burr sign (*p*<0.001), pleural depression sign (*p*<0.001), vacuole sign (*p* = 0.03), Vascular bundle sign (*p* = 0.004), bronchial sign (*p*<0.001), diameter of GGN (*p*<0.001), and CTR (*p*<0.001) (see Table 1).

**Table 1:** Single factor analysis of IA.

	Total (*n* = 556)	Prep-IA	IA	*p*-value
		*n* = 123	*n* = 433	
Age (years)	61.51 ± 26.51	60.04 ± 7.96	61.93 ± 29.73	0.486
Gender, *n* (%)				0.85
Male	212 (38.1%)	46 (37.4%)	166 (38.3%)	
Female	344 (61.9%)	77 (62.6%)	267 (61.7%)	
History of respiratory disease, *n* (%)				0.886
No	450 (80.9%)	99 (80.5%)	351 (81.1%)	
Yes	106 (19.1%)	24 (19.5%)	82 (18.9%)	
Family history, *n* (%)				0.302
No	456 (82.0%)	97 (78.9%)	359 (82.9%)	
Yes	100 (18.0%)	26 (21.1%)	74 (17.1%)	
Currently smoking, *n* (%)				0.084
No	429 (77.2%)	102 (82.9%)	327 (75.5%)	
Yes	127 (22.8%)	21 (17.1%)	106 (24.5%)	
Tumor site, *n* (%)				0.113
Upper left	165 (29.7%)	32 (26.0%)	133 (30.7%)	
Lower left	49 (8.8%)	11 (8.9%)	38 (8.8%)	
Upper right	43.7 (22.8%)	65 (52.8%)	178 (41.1%)	
Middle right	4.0 (22.8%)	5 (4.1%)	17 (3.9%)	
Lower right	13.8 (22.8%)	10 (8.1%)	67 (15.5%)	
GGN diameter	2.44 ± 0.27	2.36 ± 2.18	2.47 ± 2.79	< 0.001***
CTR	0.38 ± 0.24	0.18 ± 0.20	0.44 ± 0.21	< 0.001***
Lobulated sign, *n* (%)				< 0.001***
No	441 (79.3%)	118 (95.9%)	323 (74.6%)	
Yes	115 (20.7%)	5 (4.1%)	110 (25.4%)	
Burr sign, *n* (%)				< 0.001***
No	443 (79.7%)	117 (95.1%)	326 (75.3%)	
Yes	113 (20.3%)	6 (4.9%)	107 (24.7%)	
Pleural depression sign, *n* (%)				< 0.001***
No	260 (46.8%)	85 (69.1%)	175 (40.4%)	
Yes	296 (53.2%)	38 (30.9%)	258 (59.6%)	
Vacuole sign, *n* (%)				0.03*
No	435 (78.2%)	105 (85.4%)	330 (76.2%)	
Yes	121 (21.8%)	18 (14.6%)	103 (23.8%)	
Vascular bundle sign, *n* (%)				0.004**
No	295 (53.1%)	51 (41.5%)	244 (56.4%)	
Yes	261 (46.9%)	72 (58.5%)	189 (43.6%)	
Bronchial sign, *n* (%)				< 0.001***
No	420 (75.5%)	110 (89.4%)	310 (71.6%)	
Yes	136 (24.5%)	13 (10.6%)	123 (28.4%)	

Data are *n*, *n* (%). ^*^p<0.05, ^**^p<0.01, ^***^p<0.001.

**Figure 3:**
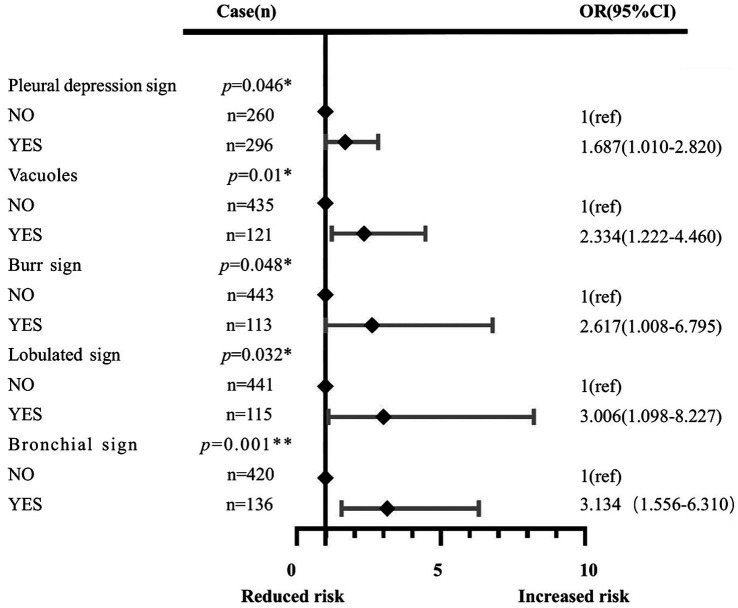
Effects of the pleural depression sign, vacuole sign, burr sign, lobulated sign, bronchial sign, diameter of GGN, and CTR. Each point represents an OR. The horizontal lines indicate 95% CI. The x-axis was based on the log scale. ORs are adjusted for the pleural depression sign, vacuole sign, burr sign, lobulated sign, bronchial sign, diameter of GGN, and CTR. *p<0.05, **p<0.01, ***p<0.001. OR: odds ratio.

The multivariate analysis was used to construct the forest map (Figure 3). We used the back-stepping method to develop the model; the final model was adjusted according to the lobulated sign, burr sign, pleural depression sign, vacuole sign, bronchial sign, diameter of GGN, and CTR. We concluded that the risk factors for the development of infiltrative stage GGN were the pleural depression sign (OR = 1.687, 95% CI 1.010–2.820), vacuole sign (OR = 2.334, 95% CI 1.222–4.460), burr sign (OR = 2.617, 95% CI 1.008–6.795), lobulated sign (OR = 3.006, 95% CI 1.098–8.227), bronchial sign (OR = 3.134, 95% CI 1.556–6.310), diameter of GGN (OR = 3.118, 95% CI 1.151–8.445), and CTR (OR = 172.517, 95% CI 48.023–619.745) (see Table 2).

**Table 2:** Risk factors for IA.

	Case (*n*)	*OR* (95% CI)	*p* value
Pleural depression sign			
No	260	1	0.046*
Yes	296	1.687 (1.010–2.820)	
Vacuole sign			
never	435	1	0.01*
<1 year	121	2.334 (1.222–4.460)	
Burr sign			
No	443	1	0.048*
Yes	113	2.617 (1.008–6.795)	
Lobulated sign			
No	441	1	0.032*
Yes	115	3.006 (1.098–8.227)	
Bronchial sign			
No	420	1	0.001**
Yes	136	3.134 (1.556–6.310)	
GGN diameter		3.118 (1.151–8.445)	0.025*
CTR		172.517 (48.023–619.745)	<0.001***

Adjusted for the model, the risk of IA was associated with the pleural depression sign, vacuole sign, burr sign, lobulated sign, bronchial sign, GGN diameter, and CTR. ^*^p < 0.05, ^**^p < 0.01, ^***^p < 0.001.

**Figure 4:**
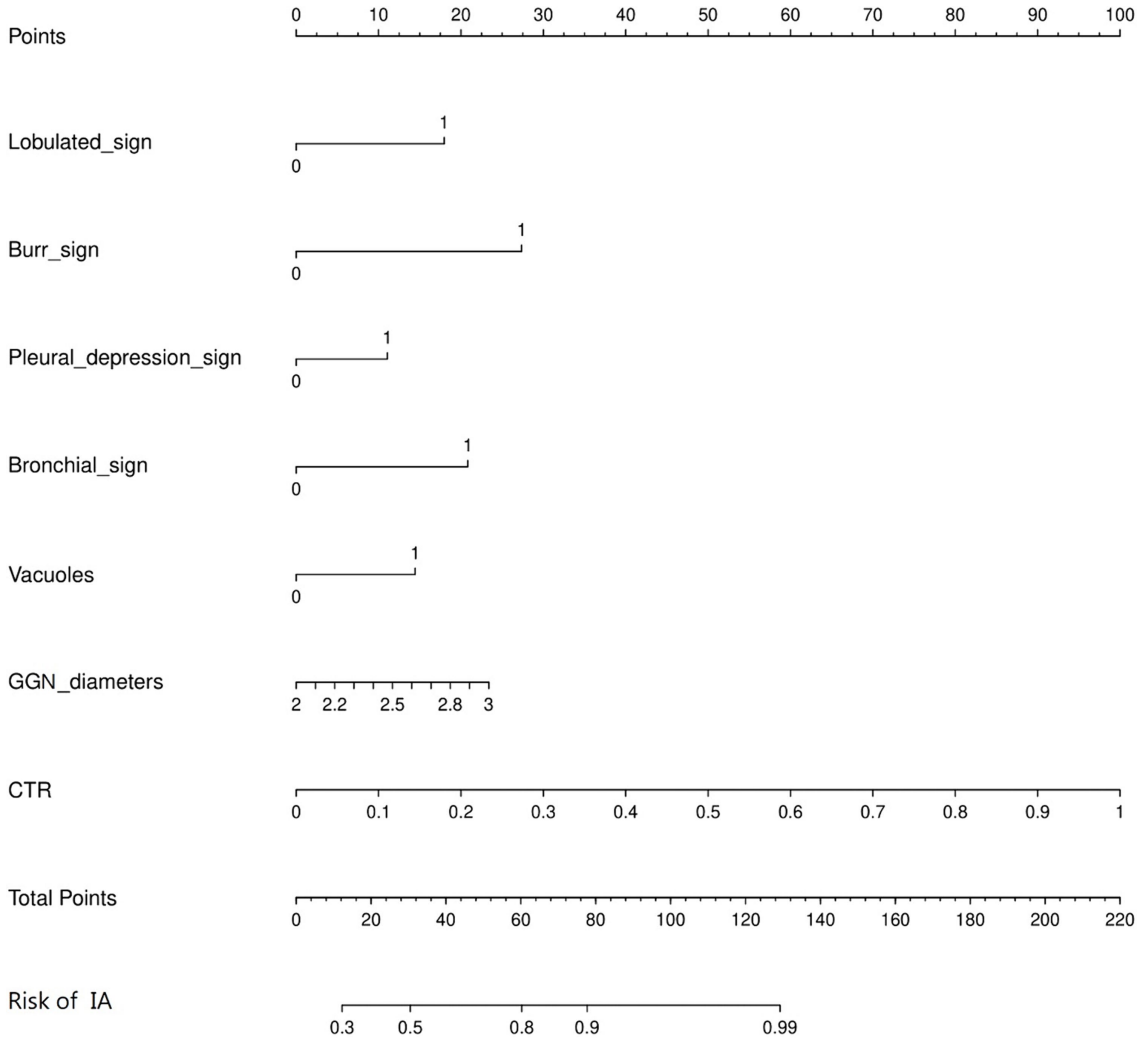
Nomogram for the prediction of 2-3 cm GGN during the infiltrative stage of the lung adenocarcinoma development. A nomogram was constructed based on the data of logistic analysis. The points of each feature were added to obtain the total points, and a vertical line was drawn on the total points to obtain the corresponding risk of 2–3 cm GGN during the infiltrative stage of the lung adenocarcinoma development.

**Figure 5:**
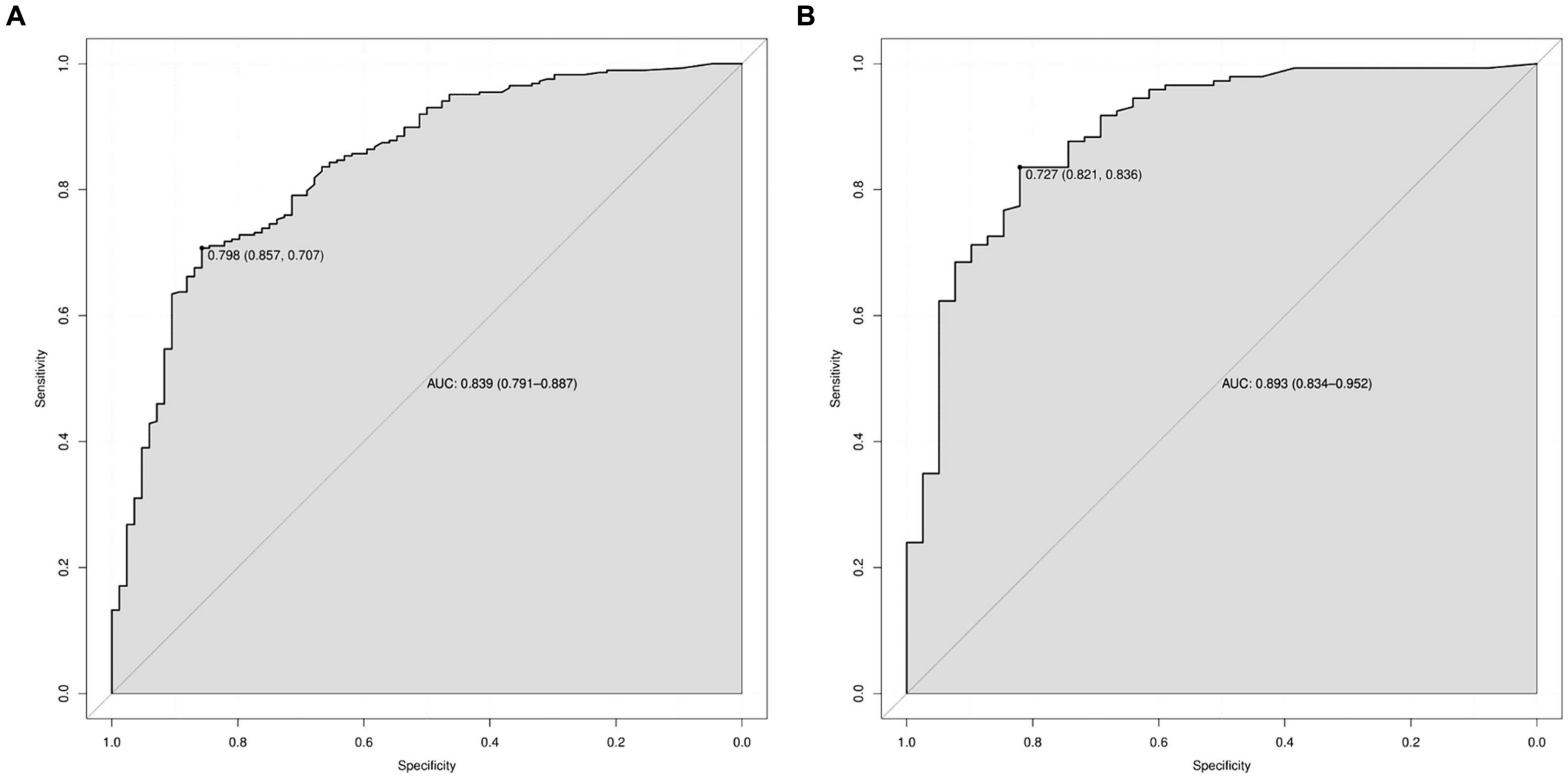
ROC curves for the prediction of 2–3 cm GGN during the infiltrative stage of the lung adenocarcinoma development in the training set and the validation set. **(A)** ROC curves of the factors and nomogram in the training set. **(B)** ROC curves of the factors and nomogram in the validation set. ROC: Receiver operating characteristic.

**Figure 6:**
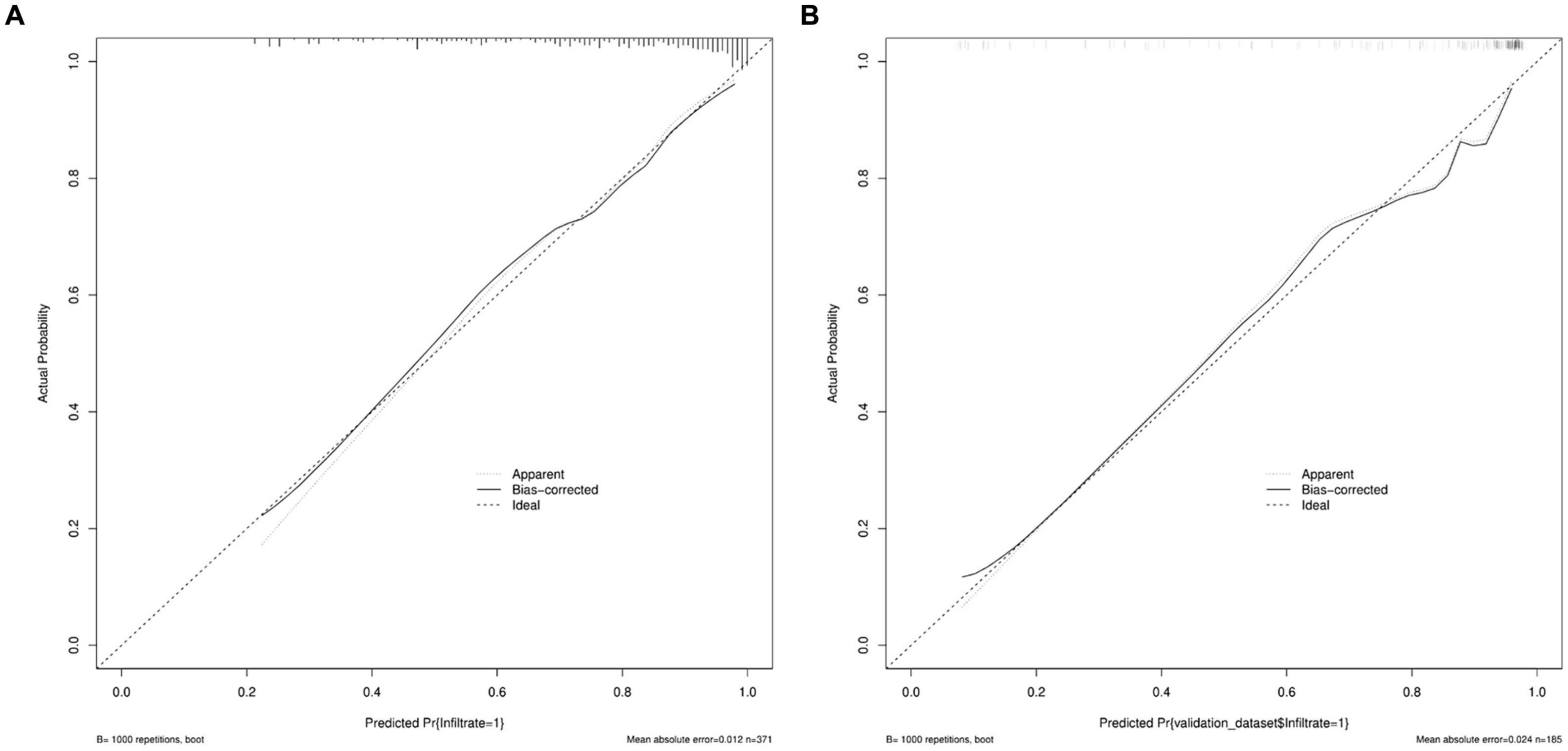
Calibration curves of nomogram prediction in the training set and the validation set. **(A)** Calibration curves of nomogram prediction in the training set. **(B)** Calibration curves of nomogram prediction in the validation set.

A predictive model of 2–3 cm ground-glass nodules developing into invasive lung adenocarcinoma was established according to the results of multivariate logistic analysis (Figure 4). The results showed that the AUC values of the training set and the validation set were 0.839 and 0.893 (Figure 5). Drawing the Calibration Curve shows that Y is close to the X-line, and the accuracy of the model is satisfactory (Figure 6).

**Figure 7:**
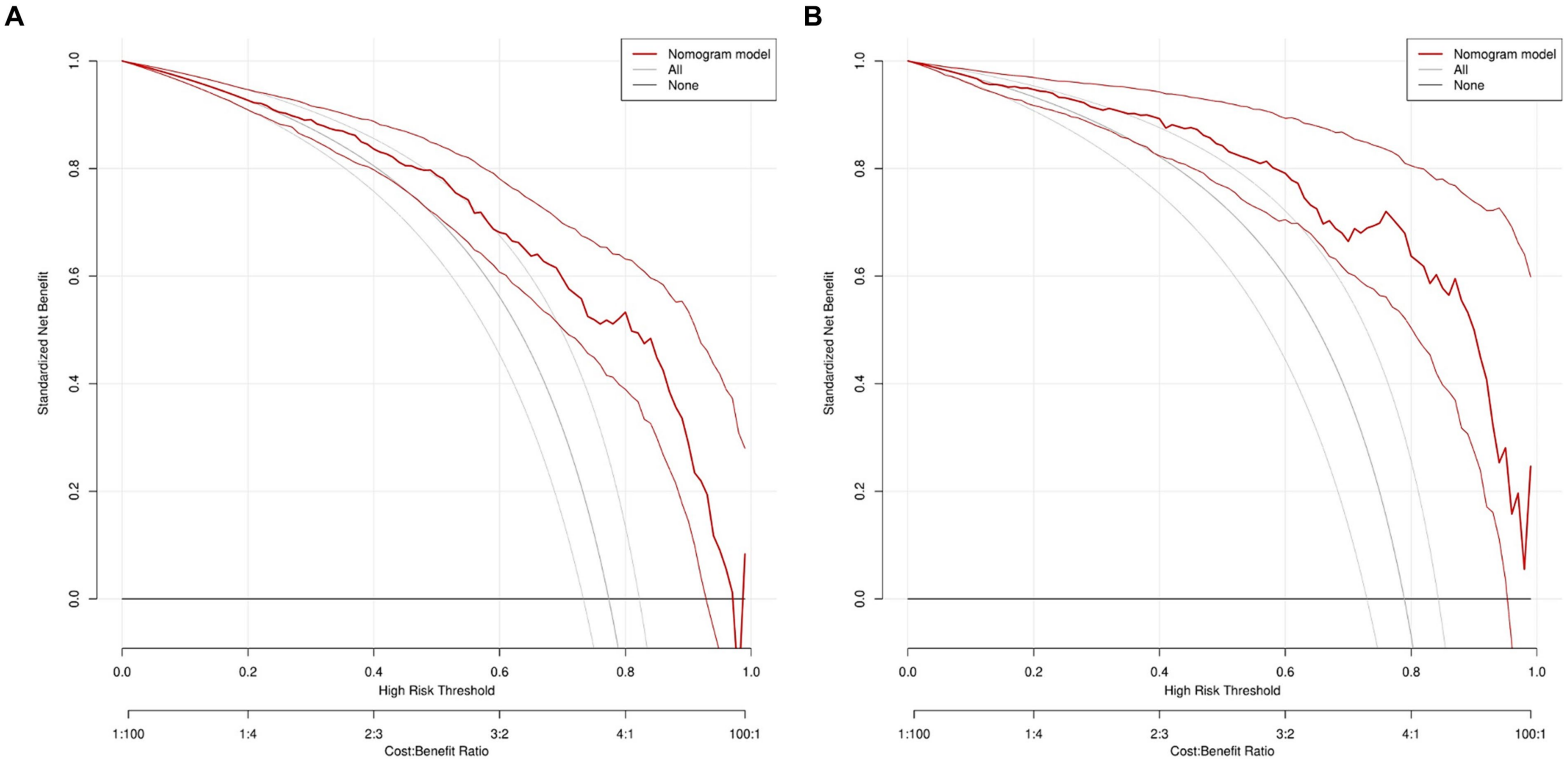
DCA of nomogram prediction in the training set and the validation set. **(A)** DCA of nomogram prediction in the training set. **(B)** DCA of nomogram prediction in the validation set. DCA: Decision curve analysis.

The DCA curve of the model was drawn with the standardized net benefit of the model as the longitudinal coordinate and the high risk threshold as the transverse coordinate (Figure 7), the results show that, in the threshold range of 0.0–1.0, the net benefit rate of the predictive model of 2 -3 cm ground-glass nodules developing into invasive lung adenocarcinoma was always > 0. Which was of clinical significance.

## 4 Discussion

With the development of imaging and the popularization of GGN follow-up, the risk prediction model of GGN based on high-resolution CT is increasing year by year (14). Some models have been applied to patients in the clinic, however, the prediction of GGN is not enough. Among them, McWilliams A’s model and Garau N’s model achieved high prediction accuracy with an AUC of 0.94 and 0.89, respectively (15, 16). However, their model was based on the Canadian and Nordic populations and included variables that were uncommon in the Chinese population and did not apply to the Chinese population projections. Sun Y’s and Liu A’s model was based on the Chinese population (17, 18), nevertheless, the AUC of their models was 0.77 and 0.836, respectively, which was less accurate than our prediction model. In addition, the above models were used to predict benign and malignant GGN. We know that GGN can initially confirm benign and malignant by 3-6 months observation, thus, the predictive model for benign and malignant nodules may be less meaningful than follow-up. However, our model is intended not only to determine the corresponding surgical approach but also to predict whether GGN reaches the invasive phase. In addition, according to the International Association for the Study of Lung Cancer (IASLC) lung cancer staging project (19), we know that GGN in different stages has different CTR ratios and different morphological characteristics. The CTR ratios of the studies 0804, 0802, and 1211 are used as a predictor of prognosis (11–13). There is a good consensus on the treatment of nodules smaller than 2cmGGN. However, the 2-3 cm GGN may be in the invasive stage due to the large diameter of the nodules and currently, there is a lack of relevant clinical studies. Therefore, the prediction model of the 2–3 cm GGN invasion stage has higher accuracy and can guide the choice of surgical method more effectively.

Our study found that GGN diameter and CTR were important predictors, and nodule diameter was the first predictor to emerge in a predictive model for the differential diagnosis of benign and malignant pulmonary nodules, using the Mayo Clinic model (AUC = 0.833) developed by Swensen et al. (20). Gould et al. built a model using data from the Department of Veterans Affairs (AUC = 0.79) (21), and Li et al. built a model developed by the People’s Hospital of Peking University (AUC = 0.89). All used the nodule diameter as a key molecule in predicting benign and malignant nodules (22). The CTR values are derived from unique imaging features of the lung. The clinical implications of this concept are supported by multiple studies (23, 24). The TNM staging guidelines for lung cancer tell us that histologically, the ground-glass component of lung nodules is associated with a lymphocyte growth pattern, while the solid component is associated with an invasive adenocarcinoma pattern (19). Some studies have found that the malignant degree of tumors increases significantly with the increase of the diameter of nodules and CTR. From the observation of patients’ survival time, the 5-year survival rate of patients with CTR less than 0.75 is 97.4%, and patients with CTR greater than 0.75 is 86.1% (25). Thus, these two indicators play a crucial role in the prediction of whether tumors reach the invasive stage, and the previous studies agree with our results.

The pleural depression sign is an imaging feature in which a subpleural nodule or tumor contacts the visceral pleura, causing the visceral pleura to be pulled toward the lesion (26). The pleural depression sign has a variety of manifestations (27) and is associated with the invasiveness of adenocarcinoma of the lung (28). Pleural depression has been studied as evidence of non-small-cell lung carcinoma infiltration into the pleura (29). Through our study, we can identify this sign as an imaging risk factor for the development of GGN in the invasive phase. Furthermore, we understand the relationship between the pleural depression sign and the pathological components of adjacent pleural regions that can promote the development of GGN-personalized treatment.

The vacuole sign is related to the infiltration and the development of GGN. A vacuole is a residual cavity formed by lung necrosis and liquefied material discharged through the bronchus. Vacuolar features are important in distinguishing lung cancer from benign lesions (30). Based on the CT findings of the solitary pulmonary nodule, Shi et al. found that the vacuole was a risk factor for malignancy, while calcification and satellite lesions were protective factors. Furthermore, a vacuole sign is the first imaging sign in the development of a tumor (31). A study found 5-year survival rates of 68.42 and 59.46% in patients with homogeneous and vacuolar nodules, respectively (32), demonstrating that the vacuolar sign has a negative impact on patient outcomes.

The burr sign was pathologically associated with increased lobular interstitial thickness, fibrotic or carcinomatous lymphatics due to small peripheral vessel occlusion (33). In this study, the spiculated sign was found to occur during the invasive development stage of GGN, this is consistent with previous studies showing that burr-bearing nodules are more likely to be malignant than those with a smooth, well-defined margin (34), and another study suggests that the positive predictive value of burr for malignancy was as high as 90% (35). In addition, classic predictive models, such as the Mayo Clinic Model (20) and Brock Model (15), also identified the spiculated sign as a risk factor for malignant pulmonary nodules.

The lobulated sign is closely associated with the growth pattern of malignant tumors, with unbalanced growth of solid components within it, resulting in radiographic changes similar to cauliflower (36). In partially solid nodules, a lobulated border is more invasive (37, 38), and this phenomenon is present in multiple cancers (39). In this study, the OR of the lobulated sign was 3.006 (95% CI: 1.098–8.227), which we hypothesize to have some predictive value in predicting the invasive development of lung cancer, and we need a larger sample to confirm this view.

The bronchial sign refers to the presence of an air-bearing track within the nodule (40), which is common in malignant nodules (41), and is mostly because the trachea extends in reverse within the tumor when the tumor retracts due to fibrosis. This symptom is seen in all lung cancer cell types but is more common in adenocarcinoma (42). According to Qiang et al., there are five types of bronchial signs: continuous open type, enveloping type, tree-like narrowing type, compressed narrowing type, and compressed flat type. The first three types are associated with the malignant progression of tumors (43). Multidisciplinary studies in imaging and molecular biology have shown that the bronchial signs are associated with mutations in epidermal growth factor receptor (EGFR) activity (44). Therefore, our study confirms that it is significant to classify the bronchial signs as an imaging risk factor for the development of infiltration in GGN.

Our prediction model has some limitations. First, our data came from a single center and were investigated only in the Chinese population, thus limiting the generalizability of the model. Furthermore, our study used a 2-dimensional CTR value rather than a 3-dimensional proportion of solid components. In addition, the characterization of the patient’s imaging features is subjective and may have an impact on the outcome. We assume that adding a three-dimensional proportion of solid components and conducting a multi-center study may improve the model’s predictive performance.

## 5 Conclusion

In this study, we investigated the difference in CT imaging features of 2–3 cm GGN during the infiltrative stage of the lung adenocarcinoma development and used this analysis to establish a decision tree model to distinguish the invasive stage from the early stage. Our study found that the pleural depression sign, vacuole sign, burr sign, lobulated sign, bronchial sign, diameter of GGN, and CTR were the imaging risk factors for the development of GGN during the invasive phase. The risk prediction model for the development of 2–3 cm GGN infiltrative stage lung adenocarcinoma based on the risk factors has some clinical significance.

## Data availability statement

The original contributions presented in the study are included in the article/supplementary material, further inquiries can be directed to the corresponding author.

## Ethics statement

The studies involving humans were approved by Ethics Committee of Second Affiliated Hospital of the Air Force Medical University. The studies were conducted in accordance with the local legislation and institutional requirements. The participants provided their written informed consent to participate in this study. Written informed consent was obtained from the individual (s) for the publication of any potentially identifiable images or data included in this article.

## Author contributions

YZ: Writing – original draft, Investigation, Methodology, Project administration, Writing – review & editing. LQ: Data curation, Investigation, Methodology, Writing – original draft, Writing – review & editing. HZ: Methodology, Writing – original draft. YW: Investigation, Resources, Writing – original draft. GG: Investigation, Writing – original draft. XW: Investigation, Writing – original draft. TZ: Funding acquisition, Resources, Supervision, Writing – original draft, Writing – review & editing.
